# Tuberculosis vaccine development: Shifting focus amid increasing development challenges

**DOI:** 10.1080/21645515.2015.1040955

**Published:** 2015-06-30

**Authors:** AJ Graves, DA Hokey

**Affiliations:** Aeras; Rockville, MD USA

**Keywords:** tuberculosis, Mtb, vaccines, vaccine development, BCG, T cells

## Abstract

A new tuberculosis vaccine is needed to replace or enhance BCG, which induces variable protection against *Mycobacterium tuberculosis* pulmonary infections in adults. Development of new TB vaccine candidates is severely hampered by the lack of a correlate of immunity, unproven animal models, and limited funding opportunities. One candidate, MVA85A, recently failed to meet its efficacy endpoint goals despite promising early-phase trial data. As a result, some in the field believe we should now shift our focus away from product development and toward a research-oriented approach. Here, we outline our suggestions for this research-oriented strategy including diversification of the candidate pipeline, expanding measurements of immunity, improving pre-clinical animal models, and investing in combination pre-clinical/experimental medicine studies. As with any evolution, this change in strategy comes at a cost but may also represent an opportunity for advancing the field.

## Introduction

Tuberculosis (TB) has long been a scourge to public health. In the early 20th century, Albert Calmette and Camille Guérin attenuated a strain of *Mycobacterium bovis* for use in preventing the spread of *Mycobacterium tuberculosis* (Mtb). The resulting vaccine, Bacillus Calmette-Guérin (BCG), was first used in human vaccinations in 1921.^1^ Since that time, BCG has become the most widely used vaccine in the world.^2^ Throughout its use, BCG has a demonstrated ability to reduce the risk of severe pediatric TB disease, specifically miliary TB and TB meningitis.^3,4^ However, despite its widespread adoption, BCG has had little impact on the global TB epidemic. A number of studies have shown that BCG is variable in eliciting protection from pulmonary TB infection in different populations.^5-7^ Further, BCG is not known to protect against latent TB and is not recommended for immunocompromised patients.^8,9^

**Figure 1.**
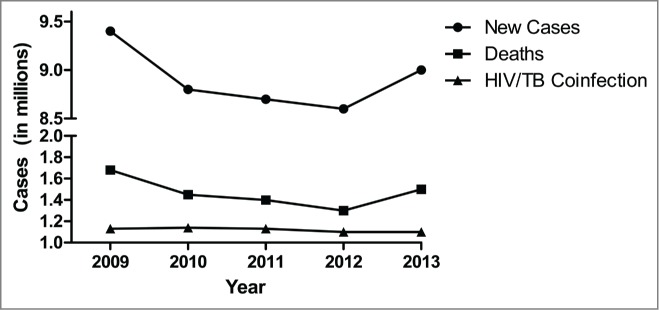
Reported new cases of tuberculosis, deaths attributed to tuberculosis, and HIV/TB coinfection cases by year. Sourced from Global Tuberculosis Reports 2010–2014 by World Health Organization.

The antibiotics isoniazid and rifampicin were introduced for fighting tuberculosis infection in the 1950′s. Mtb infections and TB disease incidence waned in developed countries where the antibiotics were available,^10^ and development of a superior TB vaccine was neglected. The global incidence of TB disease took a turn for the worse in the late 1980s and 1990s, mainly due to the spread of HIV infection and resultant immunosuppression of individuals with latent Mtb infection (LTBI), an effect particularly seen in developing countries with high incidences of both HIV and Mtb infection, such as South Africa. The incidence of multiple drug resistant (MDR) strains of TB also began to rise.^11,12^ As global travel increased, TB infections began to rebound and the World Health Organization declared tuberculosis a global public health emergency in 2005 as a result.^13,14^ In 2013, 9 million new cases of tuberculosis were reported, and 1.5 million deaths were attributed to TB.^15^ Both of these numbers represent increases compared to recent years (**Fig. 1**).^15-19^ Over 2 billion people throughout the world are infected with Mtb.^20^ Extremely drug resistant (XDR) tuberculosis has now been diagnosed in 100 countries,^15^ and incidences of totally drug resistant (TDR) tuberculosis have been reported in India, Iran, and Italy.^11,12,21-23^

Despite numerous advances in science and technology since the introduction of BCG, TB vaccine development faces a number of significant challenges. Perhaps the biggest challenge facing TB vaccine research is the lack of an immune correlate of protection. This correlate of protection represents the composition and magnitude of immune response required for protection from tuberculosis disease. Without a correlate of protection, researchers must rely on the outcomes of large scale clinical studies to demonstrate efficacy. These studies require a large sample size to show statistical significance, which means these studies carry both a high level of expense and a high level of risk. Alternatively, a correlate of protection could be a signal observed in an animal model that is predictive of vaccine-induced protection in humans. However, animal models in TB vaccine research are varied and unproven in that there is yet to be an established link between a vaccine effect observed in animal models and human protection. With these factors combined, the lack of data regarding a correlate of protection or other measures of vaccine efficacy makes it difficult and risky to manage a global pipeline of vaccine candidates.

Despite the lack of a correlate of immunity, there is extensive pre-clinical and clinical evidence to suggest that both CD4+ and CD8+ T cells, along with the Th1 cytokines IFN-γ and TNF, are vital for protection from tuberculosis.^24-35^ Armed with this knowledge, researchers set out to craft a new generation of TB vaccine candidates. The majority of these new candidates are positioned as a heterologous boost following a BCG prime administered during infancy or young adulthood. ^7,36^ These candidates also are generally focused on promoting strong T-cell immunogenicity. Several of these candidates have transitioned to clinical trials, reporting excellent safety profiles and shown to boost T-cell responses.^36^

One of the first candidates advanced to a proof-of-concept clinical study is known as MVA85A. Developed as a collaboration between the Oxford Emergent Tuberculosis Consortium and Aeras, the MVA85A candidate is a modified vaccinia Ankara virus encoding the Mtb antigen 85A.^37^ In preclinical and early phase clinical studies in adults, MVA85A was shown to induce strong CD4+ T cell responses, characterized by IFN-γ and TNF secretion,^38,39^ and modest protection was observed in animal models.^40^ MVA85A advanced to a Phase IIb clinical efficacy study, the first efficacy study in infants for tuberculosis since the 1960′s. Nearly 2,800 infants were enrolled into the study, yet the vaccine did not enhance efficacy compared to placebo and immunogenicity was generally poor.^41^ A second phase II trial for MVA85A was recently completed.^42^ This trial enrolled 650 HIV+ adults, and was again well-tolerated. The vaccine also elicited antigen 85A-specific T-cell responses. Similar to the infant trial, however, no efficacy against Mtb infection or disease was detected in this trial.

Unfortunately, the historic MVA85A efficacy trials may have provided researchers with more questions than answers. Was the low observed immunogenicity due to a flaw with the candidate, or did changing the patient population from healthy adults to infants or HIV+ adults have the most dramatic effect? Would efficacy have been improved if the magnitude of immune response was higher, or are T-cell responses alone insufficient for immunity to Mtb? Is a vaccine comprised of a single antigen sufficient for inducing protective immunity to an organism as large as Mtb?

### Shifting development strategy

Although development of TB vaccine candidates has continued, progress has slowed while researchers consider the impact of the MVA85A studies and develop strategies to address the challenges facing the field. An emerging strategy has been described as a “shift to the left” (STTL). The STTL strategy changes the focus from candidate translational development to an emphasis on preclinical and experimental medicine studies in an effort to elucidate answers critical to moving the field forward. We suggest there are 4 critical points to consider for this STTL strategy: diversification of the candidate pipeline, expanding measurements of immunity, improving animal models, and further investment into preclinical/experimental medicine studies.

### Diversification of the candidate pipeline

A recent survey of the current TB vaccine candidates in clinical trials shows 13 total candidates.^7,43^ Eight of those candidates, representing over 60% of the portfolio, rely on traditional vaccine platforms like protein/adjuvant or viral vectors like MVA or adenovirus for delivery of protein antigens. Furthermore, 6 of those 8 candidates include antigen 85. Only a handful of candidates under clinical investigation use alternative strategies such as attenuated Mtb, recombinant BCG, or whole cell mycobacterial extracts, which may contain lipid, metabolite, and glycoprotein antigens that may be critical for anti-Mtb immunity.

The current portfolio of vaccine candidates in clinical trials highlights a weakness that needs to be addressed in the STTL strategy, but will take time. Heeding evidence generated from previous studies supporting cellular immunity as a key for combating Mtb infection, the majority of candidates stimulate either CD4+ Th1 or CD8+ responses.^7^ A major problem with the the cellular immunity requirement dogma, however, is that development of vaccines driving other response types has been limited, thus constraining the diversity of candidates in the current clinical pipeline. The similarity in vaccine platforms, antigen cassettes, and induced immunogenicity duplicates effort in the current pipeline without increasing our understanding of alternative and potentially protective immune responses. Further, in an environment with limited funding opportunities, the development of many similar candidates may limit investigation into other platforms and antigens, especially when down-selection is hindered by the lack of a correlate of protection.

Moving forward, the shift in development strategy will focus on testing immune paradigms. A candidate should be able to stand as the best available candidate to stimulate a certain type of immune response, or otherwise demonstrate novelty in its mechanism of action. There are several paradigms of interest that expand the scope of vaccines well beyond traditional cellular immunity. These paradigms include: T-cell responses beyond Th1, antibody immunity, NK cell immunity, innate/adaptive immune system interactions, alternative antigen approaches such as lipids and metabolites, and immune compartmentalization. While investigation into the paradigm itself may provide new information regarding immunity to Mtb, the paradigm will need to be further evaluated for the relevant clinical effect, in this case protection from infection or disease.

Although studies have shown CD4+ Th1 and CD8+ cellular immune responses are significant for fighting Mtb infection, these responses may be insufficient on their own for protection. Recent studies have implicated possible roles for Th17^44-46^ and Th22^47^ CD4+ cells in Mtb immunity, as have non-traditional T cells like MAIT^48,49^ and γδ T cells.^50^ Currently, none of the candidates in the clinical portfolio have been designed to drive those specific responses, and those responses have not been a focus of immunogenicity evaluations or relevant biological effect. The DNA vaccine platform, which is currently not represented in the clinical TB vaccine candidate portfolio, is an example of an emerging technology that could diversify the available candidates. Through the inclusion of molecularly-encoded adjuvants, DNA vaccines may be able to direct immune responses in a particular direction of interest.^51^

Given the wealth of evidence supporting the role of cellular immunity response to Mtb infection, antibody-based immunity has been largely abandoned in terms of vaccine candidate design. Antibodies, however, may have a number of potential roles in the response to Mtb infection. These roles may include opsonization for antibody-dependent cell-mediated cytotoxicity (ADCC), modulation of the T-cell response, or neutralization of mycobacteria for limiting spread of infection.^52^ The utility of antibodies is a source of skepticism within the field,^53^ but remains a paradigm worth investigating in the preclinical and clinical arenas.

Natural killer (NK) cells are another cell type that has been implicated in tuberculosis immunity.^54^ Though initially thought of as a member of the innate immune system, NK cells have been shown to have memory-like properties. Stimulation of NK cell responses represents a potential avenue for anti-Mtb immunity, but is thus far not represented in the clinical candidate portfolio.

In a related paradigm, evidence is emerging of a greater connection between the adaptive and innate immune systems than previously was understood. Specifically, it appears that the adaptive immune system has a capacity for modulating innate responses, including interactions between innate cells and antibodies, chemokines, and cytokines.^55^ IL-17, for example, directs inflammation and a greater innate response to infection.^56^ Although data is still emerging, it appears more and more as though the immune system is an interrelated ecosystem reliant on and affected by multiple interactions rather than a simple branching system.

Another paradigm worth investigating is non-traditional antigens. The current crop of candidates relies heavily on protein-based antigen presentation for immune responses, although whole cell mycobacteria vaccine platforms may allow for recognition of alternate antigens. Roles for lipids^57^ and mycobacterial metabolites^48^ in triggering anti-Mtb responses have been demonstrated. More work is needed to understand the role of lipids and metabolites in Mtb immunity and their potential as vaccine targets.

Immune compartmentalization is a vaccine strategy that has been under investigation for some time. Essentially, the strategy is founded on the assumption that lung-resident immune memory may be beneficial for combating lung infections such as Mtb. This approach utilizes lung vaccination, such as via aerosol, to harness immune imprinting for targeting cells back to the lungs rather than a typical intramuscular vaccine that relies on circulating lymphocytes migrating to the site of infection. Safety remains a concern, as pulmonary pathology due to vaccine response is seen as a major risk. This strategy has been examined in pre-clinical studies^58,59^ and tested in recent clinical trials.^60^

These paradigms are underrepresented or absent in the current clinical candidate pipeline. Further investigation of these paradigms is warranted, but all will require investment of funds, time, and effort.

### Expanding measurements of immunity

Mirroring the candidate pipeline focus on driving Th1 responses, the majority of assays for assessing clinical trial specimens also look almost exclusively at Th1 responses. Although the focus for a desired Th1 response is based on a solid foundation, other immune responses may be necessary for vaccine efficacy. Given how little is understood about the human immune system and the immune response to Mtb infections, a greater emphasis should be placed on measuring responses beyond Th1. Already, assays are being redesigned to incorporate a broader scope, examining Th17 and Th22 responses as well as the role of T cell memory.^61^ This, however, is only the beginning of the assay development that is necessary to address the paradigms outlined above. While some small scale experiments have proven concepts, assay scale-up and implementation will be critical in order to be cost-effective for evaluating clinical trial specimens. Assays will need to be improved for new cell types, adapted for *ex vivo* stimulation using lipid and metabolite antigens, and examine tissues beyond peripheral mononuclear blood cells (PBMCs).

One current challenge for measurements of immunity comes from whole-cell vaccine candidates. These candidates typically comprise of the entirety of cellular components from BCG or other mycobacteria. For vaccines of this nature, it is critical to design an assay that differentiates immunogenicity driven by BCG and immunogenicity driven by the vaccine candidate. Answering this challenge will be imperative for the assessment and development of whole-cell vaccine candidates.

Perhaps the best method to broaden our understanding of immunogenicity following vaccination is to examine gene expression through RNA sequencing technology. Samples have already been collected for RNA sequencing in a number of TB vaccine trials, but only a handful of results have been published.^62,63^ RNA sequencing is a critical method for understanding tuberculosis infection, but the expense of sequencing and interpreting conclusions from the volume of data generated are challenges that must be surmounted.

### Improving animal models

Animal models for tuberculosis research and translational development need improvement. The models employed typically do not mimic the course of infection observed in humans. For example, the widely available C57BL/6 and BALB/c mouse strains do not exhibit granuloma formation following infection as typically observed in humans.^64^ Animal challenge studies also typically employ laboratory strains of Mtb that are quite genetically dissimilar to clinical isolates like the virulent Beijing strain.^65^ Ten to 20 times more bacteria are being used for challenge than what is thought to cause infections in humans, raising further questions about the ability of current animal challenge experiments to identify vaccine candidates likely to have efficacy in humans.^66^ Genetic disparities are also a concern, such as the absence of most CD1 subtypes in mice as well as the distribution of Toll-like receptors, and create further uncertainty on how the mouse model may correlate to human infection. In some regards (like granuloma formation), NHPs more closely mimic human infection. NHP models, however, are expensive to use for immunogenicity and challenge studies and also face limitations on facilities, space, and expertise. Additionally, few models have been adopted to mimic latent infection in either mice or NHPs.^67,68^

One role for an animal challenge model is to predict vaccine efficacy. Clearly, broader efforts are needed to address the deficiencies in the animal models to increase their predictive capabilities. Work is ongoing for the creation of a natural transmission model that may address some of the concerns regarding animal challenge. With this model, animals infected by aerosol would share an air chamber with non-infected animals. The infection would spread at a slower rate, but in a manner more reflective of natural transmission. Though this natural transmission model would be a significant step forward, the model is complicated by increased costs and logistics for housing the greater number of animals in BSL-3 conditions.

Another need is to corroborate animal models against each other or, preferably, to human data through experimental medicine studies (see below). It remains unclear how a protective result in the mouse model relates to the NHP model, and whether or not the induced immunogenicity in mouse correlates to NHPs or humans in terms of composition and magnitude. However, studies in this vein will be necessary, as the NHP model is too expensive to sustain large-scale pre-clinical portfolio management and the mouse model currently has too many uncertainties.

New tools will assist the advancement of animal model research. The introduction of new monitoring tools like PET/CT scans for NHP studies^67^ and the IVIS mouse^69^ allow new opportunities for *in vivo* monitoring of disease progression. However, despite the potential usefulness of these tools, they remain expensive and are not yet widely available for multi-center studies.

### Investing in pre-clinical/experimental medicine studies

As mentioned above, one way to address diversification of the global portfolio is to invest further in preclinical development and experimental medicine studies. This type of investment will be necessary for proving the concept behind a new vaccine candidate. Ideally, a promising vaccine candidate will be tested in an NHP study, examining both immunogenicity and protection. In parallel, the candidate will be tested in a small-scale human experimental medicine study. This study will primarily examine the immune response type and magnitude generated in humans, and allow for comparison back to the NHP model. If the response types match and the NHP model demonstrated protection, there is a better indication of success in a larger-scale clinical trial. Of course, this approach assumes that human and NHP responses to Mtb infection are similar, and that the assays used in the studies are reliable. One major difference from the current product development strategy is that early phase clinical trials typically emphasize safety over immunogenicity with a focus on further clinical development. With the experimental medicine strategy, immunogenicity will play a larger role in determining the advancement of a vaccine candidate. Using the experimental medicine studies to confirm findings in pre-clinical NHP studies will be a central aspect of the change in development strategy. Further clinical studies will then be necessary to verify human protection.

Another opportunity is the creation of a safe human challenge model.^70^ A human challenge model has been helpful for the advancements being made in the field of malaria vaccines.^71-73^ Work is underway to create a human challenge model in tuberculosis using BCG,^74^ yet much work remains to corroborate this model to show how it relates to typical pulmonary infection.

### An opportunity worth seizing

The shift in strategy will be costly in terms of both money and time lost for candidate development, but will perhaps lead to more rational selection of candidates for advancement. However, current opportunities exist for the field to respond in an agile manner that will allow new ideas to emerge and be put to the test quickly. The emergence of electroporated DNA vaccines and similar technologies offer such an opportunity. Recent clinical trials for diseases such as HIV and cancer have demonstrated electroporated DNA (EP-DNA) vaccines to be safe, well-tolerated, and highly immunogenic.^51,75-77^ This research has also indicated that EP-DNA vaccines are capable of inducing broad T-cell responses (both CD4+ and CD8+) as well as antibody responses. The EP-DNA platform also demonstrates versatility in immune response modification not available with most other platforms. The ability to include molecularly-encoded cytokine adjuvants like IL-12, IL-15, and IL-23 offer the possibility to directly modulate the immune response in ways that traditional platforms cannot.^51,76,78,79^ EP-DNA has also been shown the capability to encode entire immunoglobulin chains.^80^ Newer technology like Vaccibody opens up possibilities for linking a DNA-encoded antigen cassette to homing molecules that could drive tissue-resident responses.^81^

DNA vaccine candidates also have a clear path forward to the clinic. Already, 4 DNA vaccine products are licensed for veterinary use,^51^ and human DNA vaccine trials are underway or completed for HIV,^76^ HPV,^75^ influenza,^82^ a variety of cancers,^83-85^ and more. In fact, one promising HPV vaccine candidate met its efficacy endpoint goal for preventing HPV-induced cervical cancer.^86^ Advancing a similarly designed TB vaccine candidate should follow a similar development pathway into the clinic, bypassing many of the regulatory issues that might slow or inhibit clinical development of other novel platforms that have yet to reach the clinic.

The shift in development strategy is also an opportunity for the TB vaccine field to re-evaluate and broaden collaborations. The shift in strategy still requires a foundation of sound science and good decision-making. It will be imperative that collaborators agree on important development goals, and be judicious with available funding. Moving forward, there is an opportunity for greater collaboration with the immunology field as a whole, focusing on utilizing broader expertise in the field to answer specific questions relating to TB. Novel immune mechanisms from the broader immunology field may deepen our understanding of Mtb infection, and in turn lead to new vaccine targets. Similarly, collaborative research into tuberculosis may reveal new discoveries about immune system functions and interactions.

In summation, the TB vaccine field is in a state of transition. While previous portfolio management relied heavily on a strategy of product development, breakthrough discoveries and promising candidates are few. Adoption of a strategy that is more focused on answering core questions and testing new paradigms will be essential for advancement of the field as a whole. While significant time, financial investment, and process development will be necessary for this transition, it is also an opportunity to survey the landscape, forge new collaborations, and propel the field forward.
